# Domain Metastability: A Molecular Basis for Immunoglobulin Deposition?

**DOI:** 10.1016/j.jmb.2010.04.011

**Published:** 2010-06-04

**Authors:** Andreas F.-P. Sonnen, Chao Yu, Edward J. Evans, David I. Stuart, Simon J. Davis, Robert J.C. Gilbert

**Affiliations:** 1 Division of Structural Biology, The Wellcome Trust Centre for Human Genetics, University of Oxford, Roosevelt Drive, Oxford OX3 7BN, UK; 2 Nuffield Department of Clinical Medicine and MRC Human Immunology Unit, University of Oxford, John Radcliffe Hospital, Headington, Oxford OX3 9DU, UK

**Keywords:** IgSF, Ig superfamily, TFE, 2,2,2-trifluoroethanol, PBS, phosphate-buffered saline, CTLA-4, X-ray crystallography, protein aggregation assays, electron microscopy, amyloid

## Abstract

We present the crystal structure of an immunoglobulin light-chain-like domain, CTLA-4, as a strand-swapped dimer displaying cis–trans proline isomerisation and native-like hydrogen bonding. We also show that CTLA-4 can form amyloid-like fibres and amorphous deposits explainable by the same strand swapping. Our results suggest a molecular basis for the pathological aggregation of immunoglobulin domains and why amyloid-like fibres are more often composed of homologous rather than heterologous subunits.

Immunoglobulin (Ig) light-chain protein deposition diseases arise when Ig domains form pathology-inducing aggregates; these can occur in a variety of organs but most frequently in the kidney.^1^ The molecular nature of the aggregates formed during such disease processes has yet to be determined in detail but two different kinds are known to exist: amyloid-like fibres as found in light-chain amyloidosis and amorphous aggregates as found in light-chain deposition disease.^1^ In the course of analyzing the structure and interactions of CTLA-4, which is providing important insights into avidity enhancement of regulatory signals at the T-cell surface,^2–5^ we crystallised and solved the structure of an *Escherichia coli*-expressed monomeric form of the protein (ecCTLA-4), which unexpectedly formed a misfolded dimer and both amyloid and amorphous aggregates under largely physiological conditions. Our work provides new insights into the stability of immunoglobulin folds and the process by which they form disease-inducing amyloid-like deposits.

A monomeric form of ecCTLA-4, which comprises a canonical Ig superfamily (IgSF) V-set domain, was engineered by mutating the cysteine at position 122, which forms a disulfide between the C-terminal membrane-proximal linker regions of the protein, to a serine. As expected, this construct was expressed as a monomer (see Supplementary Fig. S1), and its crystal structure revealed the characteristic V-set IgSF fold (see Fig. 1a and Table 1). Although only one molecule of CTLA-4 is present in each crystallographic asymmetric unit, an unexpected swapping of the C-terminal β-strands between adjacent ecCTLA-4 molecules results in the formation of an artificial homodimer (compare the topology diagrams in Fig. 1b and c). In Fig. 1d we show an omit map with phase information for the strand-swapped region excluded from the calculation: the 2*F*_o_ − *F*_c_ map shows clearly the strand-swapped density. The GG′ strands of one domain replace the GG′ strands of a neighbour, and *vice versa*, forming a compact dimeric unit. A structural comparison with the native, non-strand-swapped crystal structures of B7-complexed CTLA-4^3^ and mammalian-expressed CTLA-4 (maCTLA-4; Yu *et al.*, manuscript in preparation) which forms a quite different disulfide-linked dimer, revealed that cis–trans isomerisation of the prolines at positions 101 and 103 relieves peptide bond strain in ecCTLA-4 seemingly driving the strand swap of the C-terminal β-strands (Fig. 1b and c). In Supplementary Fig. S2, we show a superposition of all four previously determined CTLA-4 crystal structures—three of which describe non-strand-swapped dimers (all additionally ligand bound)^2,3,6^ and one of which is an unliganded monomer.^7^ In addition to superposition of the domains themselves, we show a close-up of the triproline loop in which cis–trans isomerisation is present in the ecCTLA-4 structure compared to the others. As well as this reconfiguration, new van der Waals and hydrogenbonding interaction networks between the amino acids forming the (opened) hinging region appear to provide stabilisation energy for the strand-swapped ecCTLA-4 dimer, alongside the formation of the native-like β-strand interactions between the GG′ and F strands.

The strand swap seen in our CTLA-4 structure is strikingly similar to that found for CD47^8^ and also that seen for a llama antibody variable heavy chain,^9^ which was hypothesised to provide a basis for understanding aggregation by Ig domains.^1^ To investigate the possibility that the strand swapping we observe could underpin additional levels of aggregation, we incubated a concentrated solution of monomeric ecCTLA-4 at room temperature for 24 h, whereupon dimeric and larger oligomeric species formed (Supplementary Fig. S3). This indicates that purified monomeric ecCTLA-4 ectodomain can refold to a relatively stable strand-swapped dimer and also produce larger aggregates. To unravel the structural basis of immunoglobulin domain aggregation, and in particular to investigate the bimodal and concurrent formation of both fibres and amorphous aggregates, we investigated ecCTLA-4 aggregation further.

To assess the amyloid-forming propensity of the monomeric and strand-swapped dimeric forms of CTLA-4, we employed an assay established by Wright *et al.* in which 2,2,2-trifluoroethanol (TFE) is used to create mildly denaturing conditions and so stimulate aggregation.^10^ Both the monomeric, refolded protein and the dimeric material produced by overnight incubation formed aggregates, the kinetics of which could be followed by monitoring the fluorescence of the amyloid-specific dye Thioflavin T. This suggests that the higher-order aggregates have an amyloid-type structure (see Fig. 2a). The rate at which aggregation occurred was comparable to that observed for other amyloidogenic proteins.^10,11^ The aggregates formed were also Congo Red-positive (see Fig. 2b), which is another indicator of amyloid-type structure. In the case of the maCTLA-4 ectodomain retaining its cysteine-containing C-terminus, the C-terminal β-strands of the homodimer appear to be prevented from strand swapping by disulfide bond formation, since this form of the protein exhibited no tendency to form aggregates, even in the presence of TFE (see Fig. 2a). This indicates that a free C-terminus, and thus also a less constrained hinging region, mediates strand swapping and amyloid-type aggregate formation. Furthermore, it implies that a fundamental metastability within the IgSF V-set fold allows strand swapping in soluble forms of CTLA-4 that is likely to be prevented by membrane anchoring and/or disulfide formation in full-length protein or the wild-type ectodomain. Aggregates of ecCTLA-4 could bind Thioflavin T (see Supplementary Fig. S4), but their capacity to do so did not increase upon additional incubation with TFE (see Fig. 2a). Thus ecCTLA-4 monomers seem to become kinetically trapped as strand-swapped dimers that, along with monomers, can then convert to thermodynamically more stable higher-order aggregates, but the aggregates themselves represent a distinct and terminally misfolded form of the protein.

What is the molecular nature of these aggregates? The ultrastructure was visualised by negative stain electron microscopy and is displayed in Fig. 2c. In addition to an apparently amorphous aggregate, individual fibres could be imaged that appear to consist of “beads-on-a-string” type structures similar to those described by Bennett *et al.*^1^ This linear, one-dimensional polymerisation would propagate as each monomer forms a strand-accepting surface (the interface that coordinates the GG′ strands in the native domain) by donating its GG′ strands to a neighbour. Consistent with such an arrangement for the CTLA-4 aggregates, circular dichroism (CD) indicates that the secondary structure of the soluble protein and that of the aggregates are very similar (Fig. 2d). The prominent spectral minimum at a wavelength of around 220 nm is characteristic for β-sheet structures and differences in the fine structure of the aggregate scan are indicative of structural rearrangement upon amyloid formation, without a change in the secondary structure. The CD spectrum of the monomer also shows that CTLA-4 was folded prior to crystallisation and that a (partially) unfolded structure, due to the presence of residual unfolding agents for instance, was not responsible for strand swapping.

When calculating the strand-swapping capacity of the IgSF domain as defined by Bennett *et al.*,^1^ it becomes clear that the monomer can only interact with two additional protomers and hence can only form a linear arrangement. As seen for sickle cell haemoglobin,^12^ the cross-β-spine of archetypal amyloid fibrils is not essential for producing large fibres that may be involved in disease manifestation, and structures with native-like folds can lead to disease phenotypes; the native fold would also be preserved in a bead-like arrangement of the CTLA-4 V-set domains in fibres, as shown schematically in Fig. 3. Similar strand swapping mediates a family of diseases known as serpinopathies in which non-amyloid-type fibres are formed.^1^

Domain swapping of the type we have observed provides an explanation for the observation made by Wright *et al.* that immunoglobulin domains of similar sequence aggregate much faster and to a greater extent into amyloid-like structures than domains of dissimilar sequence: swapping β-strands of dissimilar sequences would effect imperfect structural complementation and decreased stability.^10^ Our observations also support the proposal^1^ that domain swapping could be the molecular basis for the formation of both fibrils and aggregates in the same disease, especially when different kinds of deposits arise from the same protein. In certain lymphoproliferative disorders, immunoglobulin domains are overproduced, resulting in amorphous and fibrillar aggregates. A soluble splice-variant form of CTLA-4 that lacks the transmembrane region and membrane-proximal linker has been identified *in vivo*, being most abundant in bone marrow, blood and lymph nodes.^13^ This naturally soluble form of CTLA-4 formed dimeric species with apparently comparable stability to that of the dimer described here, as well as some higher molecular weight aggregates. In patients with autoimmune thyroid disease, the levels of expression of soluble CTLA-4 are as much as 7- to 20-fold greater than in normal individuals;^14^ furthermore, individuals suffering from myasthenia gravis,^15^ systemic lupus erythematosus^16^ and systemic sclerosis^17^ also show elevated soluble CTLA-4 expression. Indeed, in systemic sclerosis the level of CTLA-4 expression appears linked to adverse progression, and aggregated protein deposits are associated with pathology.^17^

It is noteworthy that the two-β-sheet DEBA–GFCC′C″ topology characteristic of IgSF domains appears independently in cytokine receptors, fibronectin, cadherins, transcription factors and bacterial cytosolic domains, indicating that it is among the most successful folding topologies. In spite of this, there are now four examples of IgSF domains exhibiting intrinsic metastability: CTLA-4, CD47, the llama antibody heavy chain and CD2.^8,9,18^ In the case of the CD2 dimer, a much more dramatic rearrangement is observed, in which the A, B, C and C′ strands of one polypeptide combine with the D, E, F and G strands of the other. It seems that the evolution of these proteins accommodated a trade-off between facile folding and potentially disease-causing metastability.

### Protein Data Bank accession code

Coordinates and structure factors for the ecCTLA-4 strand-swapped dimer have been deposited with Protein Data Bank accession code 2x44.

## Figures and Tables

**Fig. 1:**
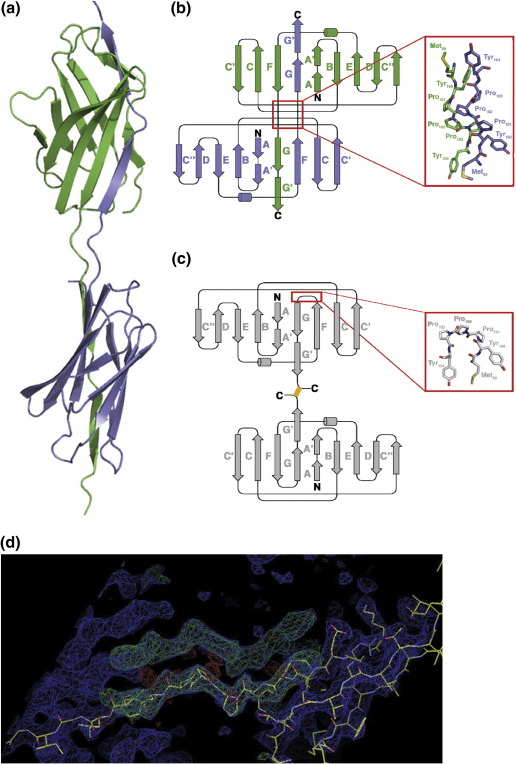
Structure of a strand-swapped Ig-domain dimer. The structure of the stand-swapped dimer is shown in (a) and its topological diagram in (b) (strands named according to standard conventions). For comparison, (c) displays the topology diagram of the homodimeric maCTLA-4 ectodomain (Yu *et al.*, manuscript in preparation). The location of the disulfide is shown in yellow. The close-ups are molecular representations of the hinge regions (red boxes) in the swapped (top, carbon in green, oxygen in red, nitrogen in blue and sulfur in yellow) and unswapped (bottom, same colours except carbon in grey) vl-Ig domains. For purification of samples, see Supplementary Fig. S1 and Ref. 19; for structure determination method and statistics for ecCTLA-4, see Table 1. (d) Omit map calculations for the structure. The hinge region of the dimer was omitted in calculation of both the 2*F*_o_ − *F*_c_ map (blue) and the *F*_o_ − *F*_c_ map (green, at + 3σ; red at − 3σ). The atomic model for one-half of the crystallographic dimer is included in the figure.

**Fig. 2:**
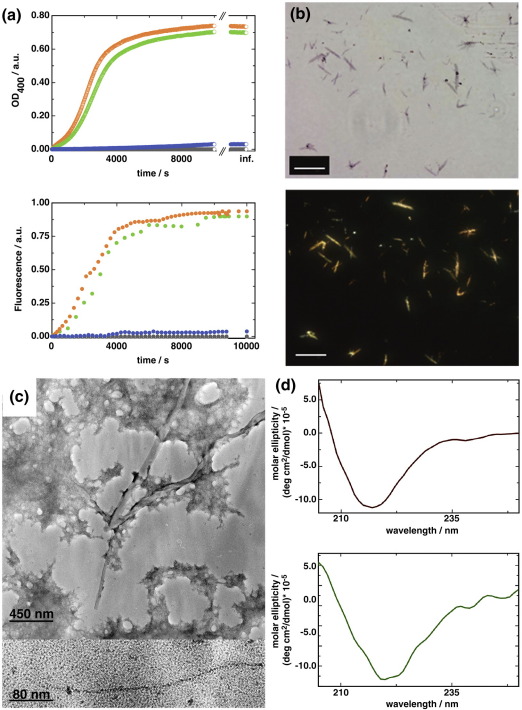
Amyloid formation by an Ig domain. (a) Monomeric CTLA4 was concentrated to 10 mg/ml and incubated for 24 h at room temperature. The solution was then applied to a size-exclusion chromatography column whereupon a stable dimeric species as well as higher molecular weight species and aggregates could be separated (see Supplementary Fig. S3a). Under the strong denaturing (but not reducing) conditions in electrospray ionization mass spectrometry only molecular weights that corresponded to the monomer were found (see Supplementary Fig. S3b, where a representative deconvoluted mass spectrum is shown). To further assay the aggregation, it was induced by incubating CTLA4 in phosphate-buffered saline (PBS) with 28% TFE and the scatter of light at 400 nm was followed in real time (see Refs. 10 and 20). Aggregate formation was followed by monitoring the scattering of 400 nm light (top) and Thioflavin T fluorescence increase (bottom) for monomer (orange), strand-swapped dimer (green), higher-order aggregate (blue) and disulfide-bonded maCTLA-4 (grey) upon incubation with TFE. Optical density (OD) and fluorescence are reported in arbitrary units (a.u.). For details of fluorescence measurements, see Supplementary Fig. S4. (b) Congo Red staining of amyloid fibrils. CTLA-4 amyloid fibrils (50 μl) were placed on a clean microscope slide and dried under air. A 10 μM solution of Congo Red in PBS at pH 7.4 was filtered several times through a 0.22-μm nucleopore filter and 50 μl was added onto the dried amyloid fibrils. A clean coverslip was placed onto the sample, which was then dried with a paper towel and sealed along the edges of the coverslip with nail varnish. The sample was imaged under normal and crossed-polarised light with a Nikon Eclipse TE2000U inverted microscope fitted with a charge-coupled device camera. Here we show aggregates imaged under bright-field conditions (top) and the same field of view with crossed-polarising filters introduced above and below the specimen (bottom). The scale bar indicates a length of 50 μm in both panels. (c) Electron microscopy of CTLA-4 aggregates. Aggregates were spun down in a benchtop centrifuge and stained with 1% uranyl acetate on carbon-coated electron microscopy copper grids. The samples were imaged on Kodak SO-163 film with a Tecnai F30 electron microscope (FEI) operating at 300 kV accelerating voltage. Here we show an electron micrograph of negatively stained aggregate; stranded structures can clearly be discerned. The close-up of an individual strand (bottom) shows the apparently bead-like arrangement of the Ig domains in the fibril. (d) Far-UV CD measurements were performed with a Chirascan spectrophotometer (Applied Photophysics) in a 1-mm path-length quartz cuvette at a concentration of 20 μM protein. Measurements were conducted in PBS (pH 7.5) for the CTLA4 before aggregate formation and after aggregate formation. The obtained ellipticities, θ, were converted to molar ellipticities, [θ], according to the equation $\left[\theta \right]=\frac{\theta }{10\times c\times l}$, where *c* is the concentration of CTLA-4 in monomer units and *l* is the path length of the cuvette. A CD spectrum of monomeric ecCTLA4 is shown (top) and a CD spectrum of ecCTLA-4 after amyloid formation (bottom). The prominent minimum around 220 nm is indicative of β-strand secondary structural elements. See Supplementary Fig. S3 for assessment of protein aggregate formation and the absence of disulfide bonds.

**Fig. 3:**
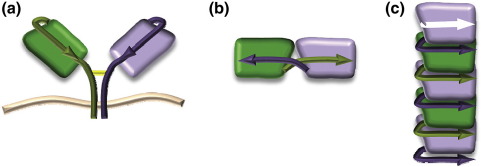
A schematic of CTLA-4 self-association. (a) Schematic of the native, membrane-bound disulfide-bonded CTLA-4 dimer on a membrane (wavy line), (b) the dimeric structure reported here (compare Fig. 1a), and (c) a possible arrangement of five subunits within a CTLA-4 amyloid fiber according to a matching strand swapping.

**Table 1:** 

	ecCTLA4
*Data collection*	
Space group	*P*3_1_21
Cell dimensions	
*a*, *b*, *c* (Å)	43.0, 43.0, 140.1
α, β, γ (°)	90, 90, 120
Resolution (Å)	18.6–2.6
*R*_merge_	3.6 (20.0)
*I*/σ*I*	29.1 (9.0)
No. of reflections/no. unique	99,136/4488
Completeness (%)	85.0 (45.8)
Redundancy	22.1 (21.3)

*Refinement*	
Resolution (Å)	18.6–2.6
No. of reflections	4289
*R*_work_/*R*_free_	19.2/24.5⁎
No. of atoms	
Protein	914
Water	50
*B*-factors	
Protein (main chain)	26.6
Protein (side chain)	31.1
Water	22.1
Overall	28.4
R.M.S.D.s	
Bond lengths (Å)	0.006
Bond angles (°)	1.03
Ramachandran plot analysis	
Outliers	0.00%
In allowed regions	4.31%
In preferred regions	95.69%

The CTLA4 vl-Ig was concentrated to 10 mg/ml in Hepes-buffered saline at pH 7.4 and crystallised in 0.02 M magnesium chloride, 0.1 M Hepes (pH 7.5), 22% (w/v) polyacrylic acid 5100 sodium salt and 0.4 M NDSB-256 (nondetergent sulfobetaine 256, dimethylbenzylammonium propane sulfonate), or with 6% 1,6-diaminohexane. Crystals appeared after 12 h and were frozen in a stream of nitrogen either with perfluorated oil or with 25% glycerol in the mother liquor as cryoprotectant. Data were collected on a MAR345 in-house detector at a wavelength of 1.542 Å. Data were indexed, integrated and scaled with the XDS package^21^ and the structure was solved by molecular replacement using PHASER^22^ with monomeric CTLA4 (Protein Data Bank code 1I8L, the structure of CTLA4 from the complex with B7-1^3^). The structure was refined with Refmac5.4^23^ using a maximum likelihood target alternated with manual rebuilding of the structure (⁎4.2% of the reflections have been used for *R*_free_ calculation, i.e., 189 reflections). Parenthetical values are for the highest-resolution shell.

## Appendix A

Figure S1 Purification of ecCTLA-4 and maCTLA-4. **A.** Gel filtration trace of refolded, purified ecCTLA-4. The absorbance was monitored at a wavelength of 280 nm and is given in arbitrary units. The inset shows an SDS PAGE gel under nonreducing conditions from the main elution peak that indicates a molecular mass around 13 kDa. M stands for molecular weight marker and C for ecCTLA-4. With minor modification the extracellular region of CTLA-4 (vl-Ig, 1-124) was purified according to Cox *et al.*, 1999) with the cysteine at amino acid position 122 mutated to a serine. Insoluble inclusion bodies were dissolved in 8 M urea, 10 mM Tris, 10 mM EDTA, 6 mM DTT at pH 8.0 and stirred at 4 °C for 2 h. Solutions were then centrifuged at 15000 × g for 30 min at 4 °C and filtered through a 0.45 μm membrane to remove any remaining aggregates. After dilution into 100 ml 3 M guanidinium hydrochloride, 10 mM sodium acetate, 5 mM EDTA, pH 4.2, the solution was slowly added in a dropwise manner to an excess of refolding buffer (50 mM Tris-HCl, 0.3 M guanidinium hydrochloride, 100 mM glycine, pH 9.5) at 4 °C. Insoluble material was removed by centrifugation at 10 000 × g and the supernatant was concentrated to 40 ml in a tangential flow ultracentrifugation cartridge (S10Y3, Amicon) with a 3 kDa cut-off. The concentrated protein was then dialysed for 3 days with two changes at 4 °C against an excess of 20 mM Tris-HCl pH 7.5, applied to a Mono Q ion exchange column (Amersham Biosciences) and eluted with an increasing concentration of sodium chloride. Monomeric CTLA-4 vl-Ig was further purified by gel filtration on a Superdex 75 10/30 column (GE Healthcare). Nonreducing and reducing SDS PAGE were conducted to monitor the purity of the fractions collected. The molecular mass of purified CTLA-4 was confirmed by electrospray ionization mass spectrometry prior to crystallization. **B.** Gel filtration trace of disulfide-bonded, homodimeric maCTLA-4. The inset shows an SDS PAGE gel under non-reducing conditions. The excised band is from the main peak and ran at a molecular weight of around 25 kDa. The purification and crystallisation of deglycosylated human CTLA-4 homodimer (maCTLA-4) will be detailed elsewhere. maCTLA-4 (extracellular domain, amino acids 1-126) was expressed as an Fc-fusion protein (CTLA-4Fc) in stably transfected CHO cells according to Yu *et al.* (manuscript in preparation). Briefly, CTLA-4Fc secreted into the tissue culture supernatant was harvested after four weeks and the protein extracted by metal-chelate chromatography using Ni-NTA agarose (QIAGEN). CTLA-4Fc was eluted from the Ni-NTA agarose with 250 mM imidazole in 20 mM Tris-HCl, 0.5 M NaCl, pH 8.0 and further purified by size-exclusion chromatography (Superdex 200 HR 10/30 column, APB). Removal of the Fc from CTLA-4Fc was achieved by cleaving the protein with thrombin in 10 mM Hepes, 150 mM NaCl, 0.05% NaN3, pH 7.4 (HBS) buffer at room temperature for 16 h. The reaction mix was then reapplied to Ni-NTA agarose to deplete the CTLA-4 of free Fc and uncleaved CTLA-4Fc. The non Ni-NTA agarose-bound CTLA-4 homodimer (maCTLA-4) was finally concentrated and buffer exchanged into HBS buffer at neutral pH for treatment with endo Hf (New England Biolabs). A contaminating fraction of endo H-resistant CTLA-4, comprising ∼ 5% of the total protein, was removed by lectin-affinity chromatography and a final gel-filtration step yielded purified preparations. Figure S2: Structural comparison to previously solved CTLA-4 structures Four CTLA-4 structures other than ours are deposited in the PDB (www.rcsb.org). In each case in the left column we show our structure, coloured as in Fig. 1, superimposed with its comparison in orange. Where a biologically significant CTLA-4 dimer was present within the crystal we show both subunits, and in each case list the accession code of the structure, in orange. 1QDT: monomeric murine CTLA-4 (Ostrov *et al.*, 2000); although dimers were found in this crystal form they are not thought to be biologically significant. 1I8L: human CTLA-4 complexed with B7-1 (Stamper *et al.*, 2001) – although the dimer-stabilising disulfide bond was mutated away (Cys > Ser mutation) in formation of this complex, a dimer thought to correspond to the native complex was found in the crystal. 1I85: human CTLA-4 complexed with a single-domain fragment of B7-2 (Schwartz *et al.*, 2001) – this dimer is a native-like disulfide-linked dimer, but the disulfide at residue 122 was disordered (this model extends to residue 120). 3BX7: human CTLA-4 complexed with an engineered lipocalin (Schönfeld *et al.*, 2009), in which the disulfide is apparent (yellow ball-and-stick representation). At the bottom we show single domains of each structure superimposed: the structure reported here in lilac, the 1DQT in red, 1IBL in blue, 1I85 in green, 1BX7 in magenta. In the central column we show the loop in which cis/trans proline isomerisation differs in the strand-swapped dimer reported here than in each of the other structures, viewed as far as the C-terminus of each model; colouring is as for the superposed single domain at the base of the left hand column. Asterisks mark an equivalent tyrosine in each structure, adjacent to the ProProPro sequence. Adjacent we show firstly in lilac the loop from the strand-swapped ecCTLA-4 structure alone, for comparison, then a superposition of all 4 deposited structure loops, then a superposition of them with our model. Figure S3: ecCTLA-4 aggregate formation in solution. **A.** Analytical size-exclusion chromatography trace of CTLA-4 incubated at 10 mg/ml for 24 h. Inset shows the calibration curve (open circles and linear fit in red). The calculated values for the three peaks predict a dimer (green), and higher molecular weight aggregate peaks (yellow and blue). **B.** A representative mass spectrum recorded under strong denaturing but not reducing conditions for the dimer peak in panel **A.** (the peak positions for the other SEC peaks were identical). Figure S4: Thioflavin T binding of monomer aggregate. Thioflavin T fluorescence emission scans monitored as a function of the wavelength showing aggregation to amyloid, after Wright *et al.*, 2005. Fluorescence was measured for 5 seconds with a Perkin Elmer LS50B fluorescence spectrometer with an excitation wavelength of 442 nm, detecting the emission at 482 nm. Excitation slits were set to 5 mm and emission slits to 7 mm. The marked fluorescence increase of the higher molecular weight aggregate (blue, from the void peak of the gel filtration trace in panel **A** of Figure S3) at an emission wavelength of 482 nm when compared to the monomer on its own before aggregation (yellow trace), underlines the formation of amyloid-like structures during aggregation.
