# Supplementary material for: Domain Metastability: A Molecular Basis for Immunoglobulin Deposition?
Source: J Mol Biol. 2010 Jun 4;399(2-2):207–13. doi: 10.1016/j.jmb.2010.04.011 (PMC2954335; doi:10.1016/j.jmb.2010.04.011)
Supplement: Figure S1 — Purification of ecCTLA-4 and maCTLA-4. A. Gel filtration trace of refolded, purified ecCTLA-4. The absorbance was monitored at a wavelength of 280 nm and is given in arbitrary units. The inset shows an SDS PAGE gel under nonreducing conditions from the main elution peak that indicates a molecular mass around 13 kDa. M stands for molecular weight marker and C for ecCTLA-4. With minor modification the extracellular region of CTLA-4 (vl-Ig, 1-124) was purified according to Cox et al., 1999) with the cysteine at amino acid position 122 mutated to a serine. Insoluble inclusion bodies were dissolved in 8 M urea, 10 mM Tris, 10 mM EDTA, 6 mM DTT at pH 8.0 and stirred at 4 °C for 2 h. Solutions were then centrifuged at 15000 × g for 30 min at 4 °C and filtered through a 0.45 μm membrane to remove any remaining aggregates. After dilution into 100 ml 3 M guanidinium hydrochloride, 10 mM sodium acetate, 5 mM EDTA, pH 4.2, the solution was slowly added in a dropwise manner to an excess of refolding buffer (50 mM Tris-HCl, 0.3 M guanidinium hydrochloride, 100 mM glycine, pH 9.5) at 4 °C. Insoluble material was removed by centrifugation at 10 000 × g and the supernatant was concentrated to 40 ml in a tangential flow ultracentrifugation cartridge (S10Y3, Amicon) with a 3 kDa cut-off. The concentrated protein was then dialysed for 3 days with two changes at 4 °C against an excess of 20 mM Tris-HCl pH 7.5, applied to a Mono Q ion exchange column (Amersham Biosciences) and eluted with an increasing concentration of sodium chloride. Monomeric CTLA-4 vl-Ig was further purified by gel filtration on a Superdex 75 10/30 column (GE Healthcare). Nonreducing and reducing SDS PAGE were conducted to monitor the purity of the fractions collected. The molecular mass of purified CTLA-4 was confirmed by electrospray ionization mass spectrometry prior to crystallization. B. Gel filtration trace of disulfide-bonded, homodimeric maCTLA-4. The inset shows an SDS PAGE gel under non-reducing conditions. [file mmc1.pdf]

Supporting Information

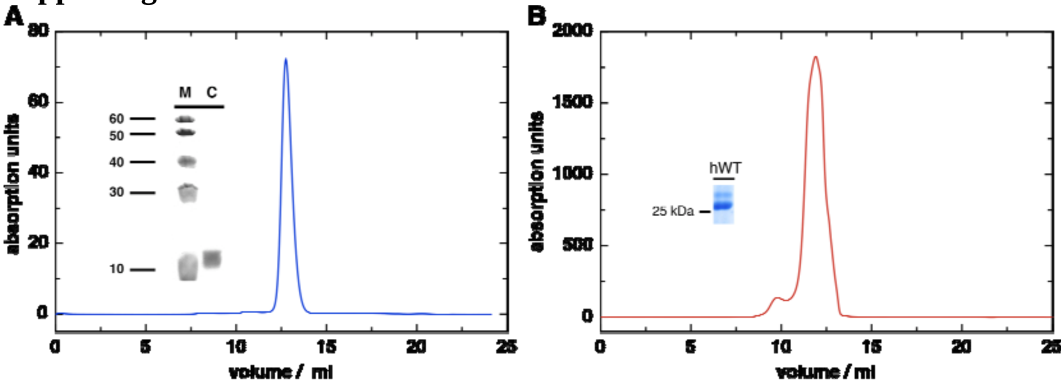

Figure S1: Purification of ecCTLA-4 and maCTLA-4.

A Gel filtration trace of refolded, purified ecCTLA-4. The absorbance was monitored at a wavelength of 280 nm and is given in arbitrary units. The inset shows an SDS PAGE gel under non-reducing conditions from the main elution peak that indicates a molecular mass around 13 kDa. M stands for molecular weight marker and C for ecCTLA-4. With minor modification the extracellular region of CTLA-4 (vl-Ig, 1-124) was purified according to Cox et al., 1999) with the cysteine at amino acid position 122 mutated to a serine. Insoluble inclusion bodies were dissolved in 8 M urea, 10 mM Tris, 10 mM EDTA, 6 mM DTT at pH 8.0 and stirred at 4 °C for 2 h. Solutions were then centrifuged at 15000 x g for 30 min at 4°C and filtered through a 0.45 μm membrane to remove any remaining aggregates. After dilution into 100 ml 3 M guadinium hydrochloride, 10 mM sodium acetate, 5 mM EDTA, pH 4.2, the solution was slowly added in a dropwise manner to an excess of refolding buffer (50 mM Tris-HCl, 0.3 M guandinium hydrochloride, 100 mM glycine, pH 9.5) at 4 °C. Insoluble material was removed by centrifugation at 10 000 x g and the supernatant was concentrated to 40 ml in a tangential flow ultracentrifugation cartridge (S10Y3, Amicon) with a 3 kDa cut-off. The concentrated protein was then dialysed for 3 days with two changes at 4 °C against an excess of 20 mM Tris-HCl pH 7.5, applied to a Mono Q ion exchange column (Amersham Biosciences) and eluted with an increasing concentration of sodium chloride. Monomeric CTLA-4 vl-Ig was further purified by gel filtration on a Superdex 75 10/30 column (GE Healthcare). Non-reducing and reducing SDS PAGE were conducted to monitor the purity of the fractions collected. The molecular mass of purified CTLA-4 was confirmed by electrospray ionization mass spectrometry prior to crystallization.

B Gel filtration trace of disulfide-bonded, homodimeric maCTLA-4. The inset shows an SDS PAGE gel under non-reducing conditions. The excised band is from the main peak and ran at a molecular weight of around 25 kDa. The purification and crystallisation of deglycosylated human CTLA-4 homodimer (maCTLA-4) will be detailed elsewhere. maCTLA-4 (extracellular domain, amino acids 1-126) was expressed as an Fc-fusion protein (CTLA-4Fc) in stably transfected CHO cells according to Yu *et al.* (manuscript in preparation). Briefly, CTLA-4Fc secreted into the tissue culture supernatant was harvested after four weeks and the protein extracted by metal-chelate chromatography using Ni-NTA agarose (QIAGEN). CTLA-4Fc was eluted from the Ni-NTA agarose with 250 mM imidazole in 20 mM Tris-HCl, 0.5 M NaCl, pH 8.0 and further purified by size-exclusion chromatography (Superdex 200 HR 10/30 column, APB). Removal of the Fc from CTLA-4Fc was achieved by cleaving the protein with thrombin in 10 mM Hepes, 150 mM NaCl, 0.05 % NaN_3, pH 7.4 (HBS) buffer at room temperature for 16 h. The reaction mix was then re-applied to Ni-NTA agarose to deplete the CTLA-4 of free Fc and uncleaved CTLA-4Fc. The non Ni-NTA agarose-bound CTLA-4 homodimer (maCTLA-4) was finally concentrated and buffer-exchanged into HBS buffer at neutral pH for treatment with endo Hf (New England Biolabs). A contaminating fraction of endo H-resistant CTLA-4, comprising ~5 % of the total protein, was removed by lectin-affinity chromatography and a final gel-filtration step yielded purified preparations.

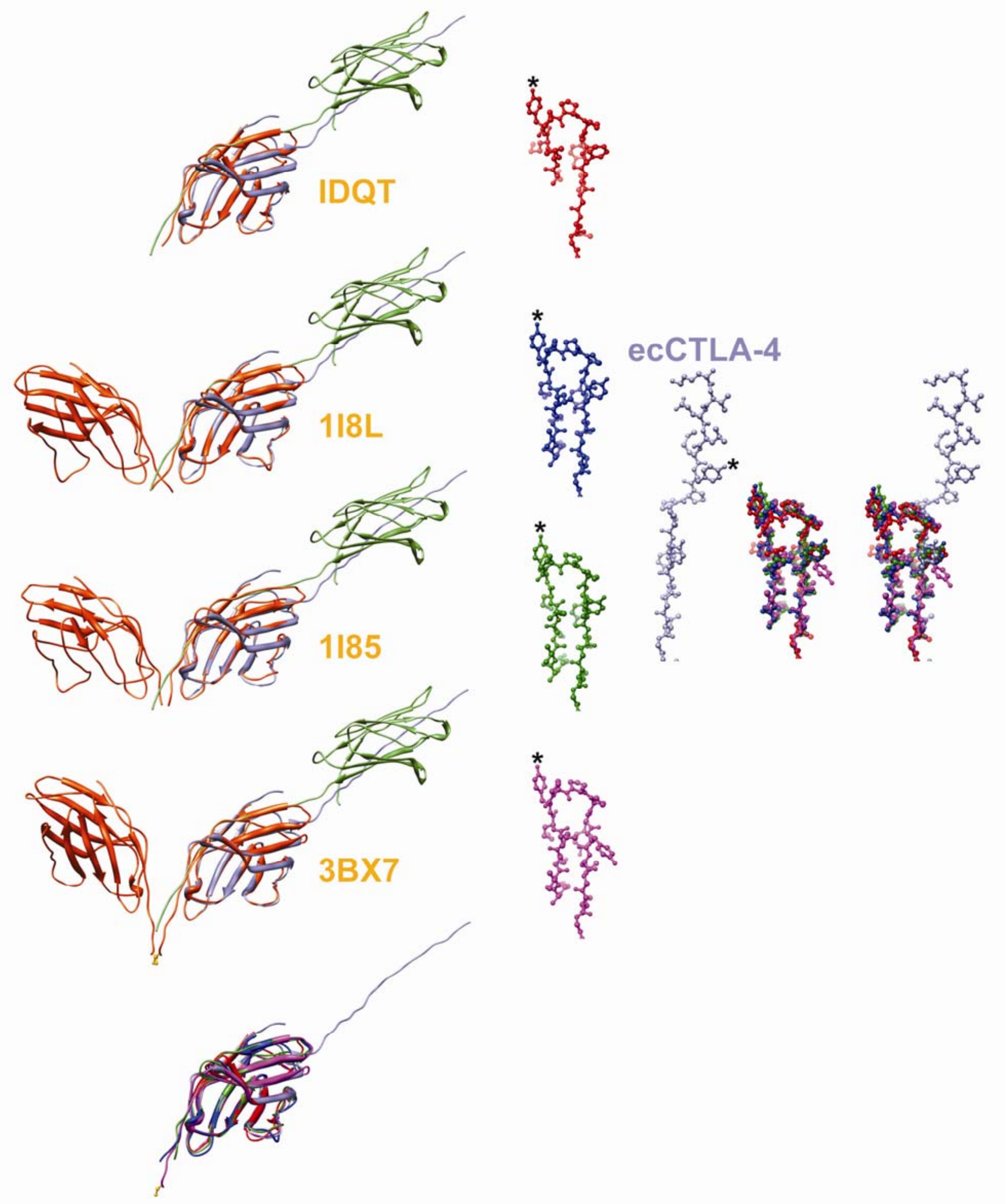

Figure S2 Structural comparison to previously-solved CTLA-4 structures

Four CTLA-4 structures other than ours are deposited in the PDB (www.rcsb.org). In each case in the left column we show our structure, coloured as in Figure 1, superimposed with its comparison in orange. Where a biologically-significant CTLA-4 dimer was present within the crystal we show both subunits, and in each case list the accession code of the structure, in orange. 1QDT: monomeric murine CTLA-4 (Ostrov et al., 2000); although dimers were found in this crystal form they are not thought to be biologically significant. 1I8L: human CTLA-4 complexed with B7-1 (Stamper et al., 2001) – although the dimer-stabilising disulphide bond was mutated away (Cys>Ser mutation) in formation of this complex, a dimer thought to correspond to the native complex was found in the crystal. 1I85: human CTLA-4 complexed with a single-domain fragment of B7-2 (Schwartz et al., 2001) – this dimer is a native-like disulphide-linked dimer, but the disulphide at

residue 122 was disordered (this model extends to residue 120). 3BX7: human CTLA-4 complexed with an engineered lipocalin (Schönfeld et al., 2009), in which the disulphide is apparent (yellow ball-and-stick representation). At the bottom we show single domains of each structure superimposed: the structure reported here in lilac, the 1DQT in red, 1IBL in blue, 1I85 in green, 1BX7 in magenta. In the central column we show the loop in which cis/trans proline isomerisation differs in the strand-swapped dimer reported here than in each of the other structures, viewed as far as the C-terminus of each model; colouring is as for the superposed single domain at the base of the left hand column. Asterisks mark an equivalent tyrosine in each structure, adjacent to the ProProPro sequence. Adjacent we show firstly in lilac the loop from the strand-swapped ecCTLA-4 structure alone, for comparison, then a superposition of all 4 deposited structure loops, then a superposition of them with our model.

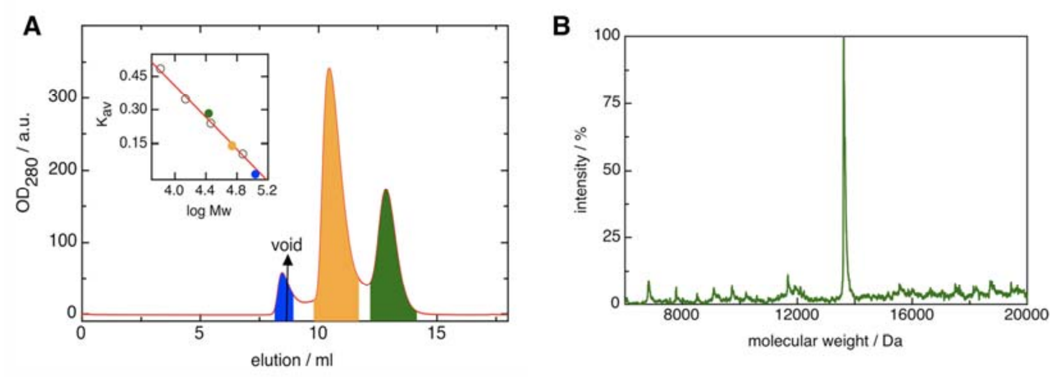

Figure S3: ecCTLA-4 aggregate formation in solution.
A Analytical size-exclusion chromatography trace of CTLA-4 incubated at 10 mg/ml for 24 h. Inset shows the calibration curve (open circles and linear fit in red). The calculated values for the three peaks predict a dimer (green), and higher molecular weight aggregate peaks (yellow and blue).
B A representative mass spectrum recorded under strong denaturing but not reducing conditions for the dimer peak in panel **A** (the peak positions for the other SEC peaks were identical).

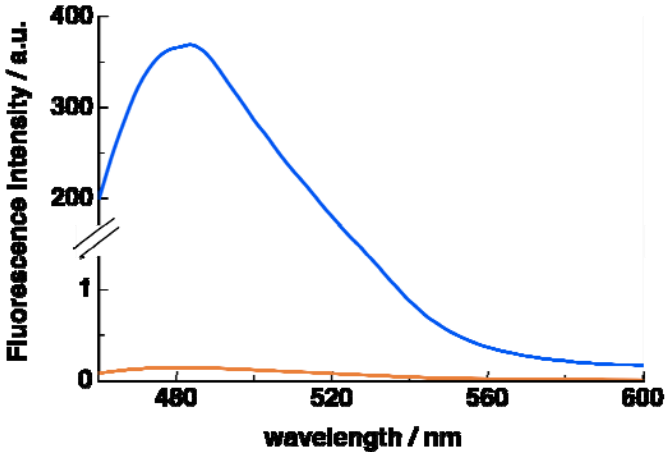

Figure S4: Thioflavin T binding of monomer aggregate.
Thioflavin T fluorescence emission scans monitored as a function of the wavelength showing aggregation to amyloid, after Wright et al., 2005. Fluorescence was measured for 5 seconds with a Perkin Elmer LS50B fluorescence spectrometer with an excitation wavelength of 442 nm, detecting the emission at 482 nm. Excitation slits were set to 5 mm and emission slits to 7 mm. The marked fluorescence increase of the higher molecular weight aggregate (blue, from the void peak of the gel filtration trace in panel **A** of **Figure S3**) at an emission wavelength of 482 nm when compared to the monomer on its own before aggregation (yellow trace), underlines the formation of amyloid-like structures during aggregation.

References

Cox, G.N., Pratt, D., Smith, D., McDermott, M.J. and Vanderslice, R.W. (1999) Refolding and characterisation of recombinant human CTLA-4 expressed in Escherichia coli. Protein. Expr. Purif. 17, 26-32.

Ostrov, D.A., Shi, W., Schwartz, J.C., Almo, S.C. and Nathenson, S.G. (2000) Structure of murine CTLA-4 and its role in modulating T cell responsiveness. Science 290, 816-819.

Schönfeld, D., Matschiner, G., Chatrwell, L., Trentmann, S., Gille, H., Hülsmeyer, M., Brown, N., Kaye, P.M., Schlehuber, S., Hohlebaum, A.M. and Skerra, A. (2009) An engineered lipocalin specific for CTLA-4 reveals a combining site with structural and conformational features similar to antibodies. Proc. Nat'l. Acad. Sci USA 106, 8198-8203.

Schwartz, J.C., Zhang, X., Fedorov, A.A., Nathenson, S.G. and Almo, S.C. (2001) Structural basis for co-stimulation by the human CTLA-4/B7-2 complex. Nature 410, 604-608.

Stamper, C.C., Zhang, Y., Tobin, J.F., Erbe, D.V., Ikemizu, S., Davis, S.J., Stahl, M.L., Seehra, J., Somers, W.S. and Mosyak, L. (2001) Crystal structure of the B7-1/CTLA-4 complex that inhibits human immune responses. Nature 410, 608-611.

Wright, C.F., Teichmann, S.A., Clarke, J. and Dobson, C.M. (2005) The importance of sequence diversity in the aggregation and evolution of proteins. Nature 438, 878-881.
